# Fabrication of Gentamicin-Loaded Hydroxyapatite/Collagen Bone-Like Nanocomposite for Anti-Infection Bone Void Fillers

**DOI:** 10.3390/ijms21020551

**Published:** 2020-01-15

**Authors:** Sho Oshima, Taira Sato, Michiyo Honda, Yasushi Suetsugu, Kazuhide Ozeki, Masanori Kikuchi

**Affiliations:** 1 Major in Industrial Science, Graduate School of Science and Engineering, Ibaraki University, 4-12-1 Nakanarusawa, Hitachi, Ibaraki 316-8511, Japan; oshima@jcoal.or.jp; 2 Bioceramics Group, Research Center for Functional Materials, National Institute for Materials Science, 1-1 Namiki, Tsukuba, Ibaraki 305-0044, Japan; tairas@meiji.ac.jp (T.S.); SUETSUGU.Yasushi@nims.go.jp (Y.S.); 3 Department of Applied Chemistry, Graduate School of Science and Technology, Meiji University, 1-1-1 Higashimita, Tama, Kawasaki, Kanagawa 214-8571, Japan; 4 Department of Applied Chemistry, School of Science and Technology, Meiji University, 1-1-1 Higashimita, Tama, Kawasaki, Kanagawa 214-8571, Japan; michiyoh@meiji.ac.jp; 5 Major in Mechanical Systems Engineering, Graduate School of Science and Engineering, Ibaraki University, 4-12-1 Nakanarusawa, Hitachi, Ibaraki 316-8511, Japan; kazuhide.ozeki.365@vc.ibaraki.ac.jp

**Keywords:** antibiotic-impregnated biomaterial, hydroxyapatite/collagen bone-like nanocomposite, gentamicin sulfate, bone void filler, antibacterial activity

## Abstract

A gentamicin-loaded hydroxyapatite/collagen bone-like nanocomposite (GNT-HAp/Col) was fabricated and evaluated for its absorption–desorption properties, antibacterial efficacy, and cytotoxicity. The hydroxyapatite/collagen bone-like nanocomposite (HAp/Col) powder was mixed with gentamicin sulfate (GNT) in phosphate-buffered saline (PBS) at room temperature. After 6 h mixing, the GNT adsorption in all conditions reached plateau by Langmuir’s isotherm, and maximum GNT adsorption amount was 34 ± 7 μg in 250 μg/mL GNT solution. Saturated GNT-loaded HAp/Col powder of 100 mg was soaked in 10 mL of PBS at 37 °C and released all GNT in 3 days. A shaking culture method for a GNT extraction from the GNT-HAp/Col and an inhibition zone assay for the GNT-HAp/Col compact showed antibacterial efficacy to *Escherichia coli* (*E. coli*) at least for 2 days. From the release profile of the GNT from the GNT-HAp/Col powder, antibacterial efficacy would affect *E. coli* at least for 3 days. Further, no cytotoxicities were observed on MG-63 cells. Thus, the GNT-HAp/Col is a good candidate of bioresorbable anti-infection bone void fillers by prevention initial infections, which is the primary cause of implant-associated infection even for rapid bioresorbable materials.

## 1. Introduction

Mainstream ceramic bone void fillers are osteoconductive in nature, and research trends are moving towards the improvement of other biological activities, osteogenic activity, and biodegradability including bioresorbability and antibacterial activity by controlling chemical composition and embedding of biofunctional molecules, including drugs. The addition of antibacterial activity is one of the important issues with regards to the fillers in terms of preventing infection after their implantation (implant-associated infection, IAI) that are increasing in developing countries and cause severe disease. The greatest cause of IAIs is biofilm formation on the surface of implants [1] and resisting immune reaction [2]. The general method to prevent infections including IAI is intravenous injection of antibiotics [3]; however, thorough dose control is necessary to reduce side-effects and appearance of drug-resistant bacteria. Thus, local delivery of antibiotics from the fillers has been studied to overcome the aforementioned risks. At least two antibacterial biomaterials for artificial joint surgery are on the market and are practically used. One is a gentamicin sulfate (GNT) impregnated polymethylmethacrylate (PMMA) bone cement, Palacos^®^ bone cement (Zimmer Inc., USA), developed according to the report of Wahlig and Buchholz [4] in 1972, and its gradual and local release of GNT reduces risks of occurrence of drug-resistant bacteria [5] and medical costs. The other is a hip-joint coated with hydroxyapatite (HAp, Ca_5_(PO_4_)_3_OH) and Ag, Ag-PROTEX^®^ ( KYOCERA Corporation, Japan), and gradual release of Ag ions inhibiting bacteria proliferation for up to 7 days. However, no bone void fillers equipped with antibacterial properties have been commercialized, despite their study by some researchers [5,6].

We focused on biodegradability as an important factor for antibacterial bone void fillers, as it reduces the risk of biofilm formation on the surface of the fillers, possible scaffolds for bacteria. Therefore, HAp ceramic-based bone void fillers are not suitable. In addition, ceramic HAp and β-tricalcium phosphate (TCP, Ca_3_(PO_4_)_2_) have lower specific surface area compared with wet-prepared materials; thus, they are not suitable for embedding sufficient amounts of antibacterial drugs.

In contrast, nanocomposites of HAp and collagen are already used practically. One is the HEALOS^®^ (DePuy Spine Inc., USA), and the other is the ReFit^®^ (HOYA Technosurgical Co., Japan). HEALOS^®^ is collagen-coated with thick HAp formed using a biomimetic method, such as simulated body fluid soaking; therefore, HEALOS^®^ has no similarity to the nanostructure of bone. ReFit^®^, developed and reported by us as a “hydroxyapatite/collagen bone-like nanocomposite (HAp/Col)”, is a self-organized nanocomposite of HAp and collagen (Col) having bone-like chemical composition and oriented nanostructure of HAp nanocrystals and Col fibers. According to the structure, HAp/Col would have a higher specific surface area than conventional ceramics and may have numerous adsorption sites for antibiotics/drugs on both HAp and Col. In addition, the HAp/Col [7] is substituted with newly formed bone within 3–6 months by incorporation into the bone remodeling process, which is faster than other biodegradable ceramic bone void fillers [8]. These properties are suitable for antibacterial bone void fillers for prophylaxis of IAIs. As an antibiotic, the GNT was chosen for a model antibiotic and candidate drug, because it is widely applied as an antibiotic to prevent general infections, including IAI, and has many advantages, such as low cost, broad antibacterial spectrum of action, low rate of primarily resistant pathogens, and good stability in terms of long shelf-life [9,10]. Furthermore, GNT is effective against most Gram-negative microorganisms and has a low risk of inducing resistant bacteria, as well as being able to be used for treatment of IAI by introduction into Palacos^®^.

In this study, the potential of HAp/Col powder as an antibacterial bone void filler was evaluated by measuring adsorption and desorption properties of GNT on HAp/Col powder, and the antibacterial properties and cytotoxicity of GNT-loaded HAp/Col (GNT-HAp/Col) powder.

## 2. Results

### 2.1. Characterization of HAp/Col and GNT-HAp/Col Powders

The specific surface area and mean pore diameter of the HAp/Col powder by Brunauer–Emmett–Teller (BET) method were 42 ± 2 m^2^/g and 15.5 ± 0.8 nm, respectively. After GNT loading on HAp/Col powder, the specific surface area and mean pore diameter decreased to 36 ± 3 m^2^/g and 14.4 ± 1.0 nm, respectively.

**Figure 1:**
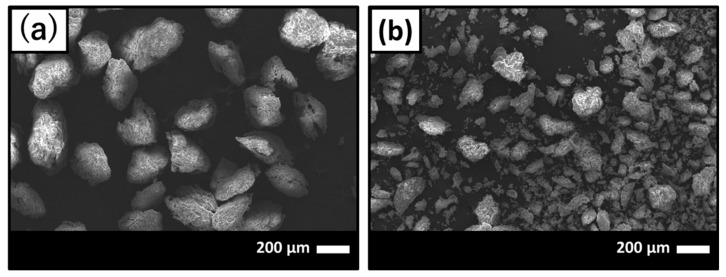
Scanning electron micrographs of (**a**) before and (**b**) after gentamicin sulfate (GNT) loading on hydroxyapatite (HAp)/collagen (Col) powder.

Scanning electronic microscope (SEM) images of before and after GNT loading on HAp/Col powder are shown in Figure 1a,b, respectively. After GNT loading on HAp/Col powder, finer HAp/Col powder was observed on SEM images (Figure 1b) around 30–40 μm in diameter because the HAp/Col powder was assumed to be pulverized original HAp/Col powder as a result of friction between particles and/or tube while mixing in GNT solution.

### 2.2. Adsorption of GNT on HAp/Col Powder

**Figure 2:**
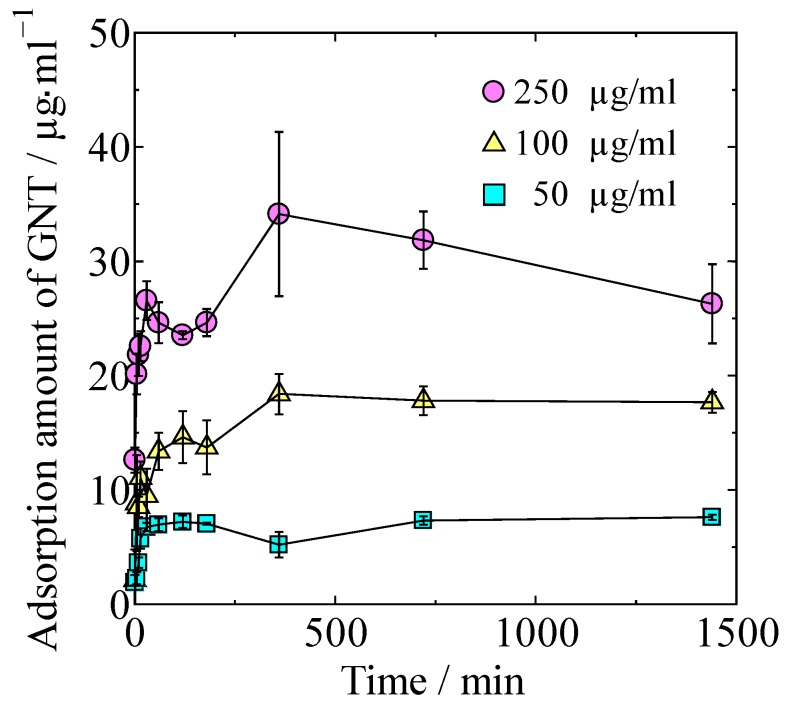
Adsorption isotherms at different concentrations of GNT.

GNT adsorption isotherms of the HAp/Col powder at different concentrations of GNT in Dulbecco’s phosphate-buffered saline (PBS) are shown in Figure 2. Adsorption amount of GNT increased with both time and concentration of GNT solution. Maximum adsorption amount of GNT in 24 h on HAp/Col powder was 3.4 ± 0.7 mg/g.

### 2.3. Desorption of GNT from Gentamicin Sulfate-Loaded HAp/Col (GNT-HAp/Col) Powder

**Figure 3:**
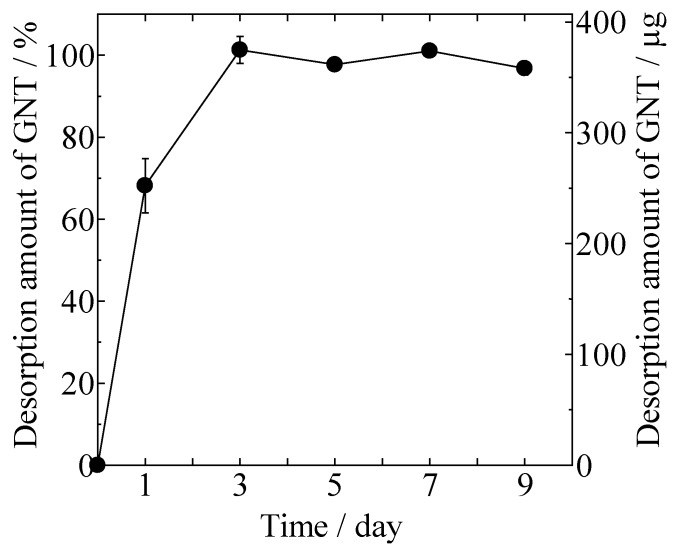
Desorption of GNT from gentamicin sulfate-loaded HAp/Col (GNT-HAp/Col) powder.

Desorption of GNT from the gentamicin sulfate-loaded HAp/Col (GNT-HAp/Col) powder increased with time, as shown in Figure 3. Approximately 68% of the GNT was desorbed at day 1, and all of the adsorbed GNT (3.8 ± 0.1 μg/mg) was desorbed within the next 2 days from 100 mg of GNT-HAp/Col powder.

### 2.4. Antibacterial Efficacy

#### 2.4.1. Shaking Culture Test

**Figure 4:**
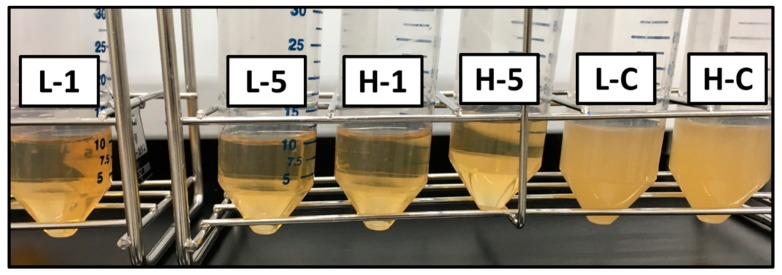
Naked-eye image of antibacterial test results by shaking culture method. L-1 and L-5 were 1 × 10^5^ CFU/mL of *Escherichia coli* with GNT extraction liquids (GNTEL) 1 and 5, respectively. H-1 and H-5 were 1 × 10^7^ CFU/mL of *E. coli* with GNTEL 1 and 5, respectively. L-C and H-C were 1 × 10^5^ and 1 × 10^7^ CFU/mL of *E. coli* with phosphate buffered saline (PBS), respectively.

Turbidity of Luria-Bertani (LB) medium after *Escherichia coli* (*E. coli,* K12W3110) culture using LB medium supplemented with the GNT extraction liquids (GNTELs) at 37 °C for 18 h is shown in Figure 4. Control cultures of *E. coli* at the lowest (L, 1 × 10^5^ CFU/mL) and highest (H, 1 × 10^7^ CFU/mL) concentrations in the JIS, as shown in Figure 4 as respective L-C and H-C, demonstrated an obvious confluent state. In contrast, *E. coli* at the L concentration was cultured in LB medium with the GNTEL 1 and 5, extractions from the GNT-HAp/Col powder for 1 or 5 days, respectively, showed bactericidal activities, noted as L-1 and L-5, respectively, in Figure 4, and bacteriostatic activities at the H concentration with GNTEL 1 and 5, respectively noted as H-1 and H-5 in Figure 4. No significant differences in antibacterial efficacy were found between GNTEL 1 and 5. The final concentration of *E. coli* is shown Table 1.

#### 2.4.2. Inhibition Zone Assay

**Figure 5:**
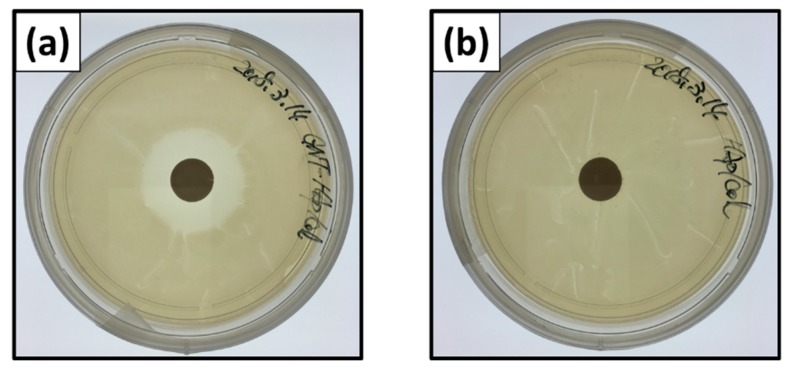
Naked-eye images of inhibition zones: GNT-HAp/Col powder compact for (**a**) 24 h, and the HAp/Col powder compact for (**b**) 24 h.

Figure 5 shows naked-eye images of inhibition zones of *E. coli* at 24 h after the incubation. Inhibition zone ratios for the GNT-HAp/Col powder compact were 4.41 ± 0.16 and 4.37 ± 0.16 for 24 (Figure 5a) and 48 h, respectively, and showed no significant difference between them. The HAp/Col compacts, however, demonstrated no inhibition zone, that is, no antibacterial activities for 24 (Figure 5b) and 48 h.

### 2.5. Cytotoxicity Test

**Figure 6:**
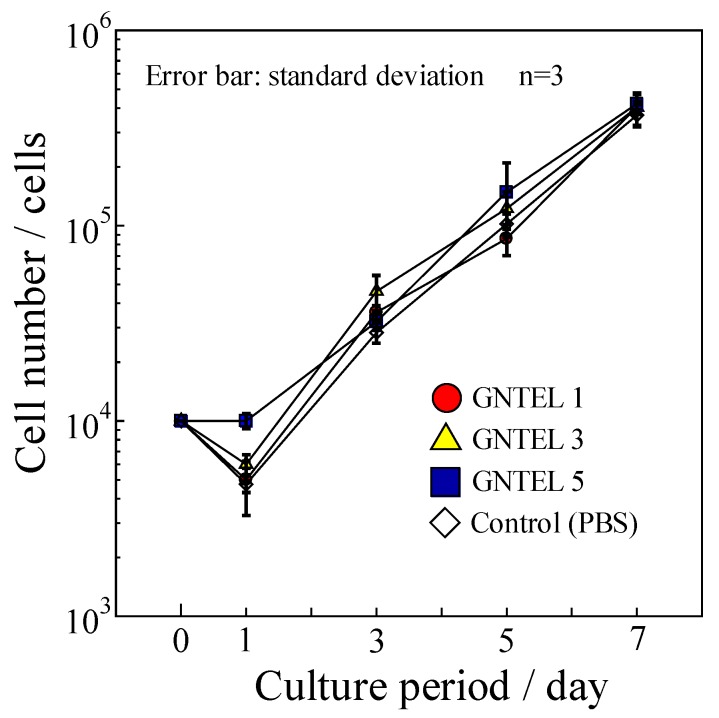
Cell proliferation curve of MG-63 using cell culture medium with GNTELs.

Proliferation curves of MG-63 using GNTELs are shown in Figure 6. No significant differences in cell numbers were found among the test and control groups at day 7. Furthermore, no influence of morphology for MG-63 cells cultured with a medium supplemented with the GNTELs was noted up to 7 days. Thus, the effective anti-bacterial concentration of GNTELs showed no cytotoxicity toward MG-63 cells.

## 3. Discussions

When the HAp/Col powder was assumed to be a dense sphere of 156 μm of mean powder diameter, the theoretical value of the specific surface area was 0.02 m^2^/g (1), where *r* is the radius of the particle. As compared with this theoretical value, the specific surface area of the original HAp/Col powder was much bigger because micro- and/or nanopores in the HAp/Col powder were formed during the freeze-drying process.

(1) $$\begin{array}{c} Specific\;surface\;area=\frac{Surface\;area\;\left[m^{2}\right]}{Density\;\left[\frac{g}{m^{3}}\right]\times Volume\;\left[m^{3}\right]}\\ =\frac{4\pi r^{2}}{\left(2.38\times 10^{6}\right)\times \frac{4}{3}\pi r^{3}}\\ =\frac{3}{\left(2.38\times 10^{6}\;\right)\times r} \end{array}$$

After the GNT-loading process, the particle size of the HAp/Col powder decreased from 100–212 µm (Figure 1a) to 30–40 µm (Figure 1b) in diameter. Obviously, the diameter decrease led to an increase in surface area; however, the above results were opposite to the size effect. Here, theoretical particle size effect for solid sphere of the HAp/Col (its density at HAp/Col mass ratio of 4:1 was approximately 2.38 g/cm^3^) was related to the reciprocal value of diameter, as shown in Equation (1). Thus, the decrease in solid HAp/Col particle diameter from 200 µm to 20 µm only increased the specific surface area from 0.013 to 0.13 m^2^/g, and was less than 0.5% against the specific surface area of the HAp/Col powder. On the other hand, adsorption of GNT molecules, less than 1 µm in size [11], on the surface of the HAp/Col powder and/or filling of some pores would be the reason for this decrease. Thus, the size effect was ignored by the effect of GNT adsorption.

The relationship between adsorbed GNT at 24 h was approximated by Langmuir’s isotherm at *R*^2^ = 0.951 instead of Freundlich (*R*^2^ = 0.868), Temkin (*R*^2^ = 0.900), Dubinin–Radushkevich (*R*^2^ = 0.859), and BET (*R*^2^ = 0.694); accordingly, the GNT adsorption on the HAp/Col powder was considered to be Langmuir-type monomolecular layer adsorption.

HAp, one of the absorbent materials, has great potential for adsorption of positively charged GNT via the PO_4_ site. On the other hand, GNT adsorption on the free amino and/or carboxyl groups in Col was reported [12]. The HAp/Col formation is explained as follows: heterogenous nucleation of HAp nanocrystals preferentially occurs on carboxyl groups of Col, and Col fibers are covered with regularly aligned HAp nanocrystals to the fibers [13]. Therefore, adsorption sites of the positively charged GNT on the HAp/Col powder would be limited by its nanostructure, negatively charged *c*-plane on the HAp, and carboxyl group on the Col that are not covered by one-another. On the other hand, Guo, Y. J et al. [10] reported that HAp particles with a specific surface area of 8.1 m^2^/g adsorbed almost the same amount of GNT as the present HAp/Col powder. A five times greater specific surface area of HAp/Col showed no increase in GNT adsorption, as coverage of adsorption sites by HAp and Col in the HAp/Col compensated for the increasing effect of the specific surface area.

For desorption of GNT from GNT-HAp/Col powder, burst release is not suited to long-term drug release for curing diseases; however, for anti-infection activity, desorption of GNT has been considered as sufficient for preventing infection according to a report showcasing that 1 g of SEPTOPAL^®^ chain releases 2–3 mg of GNT in the same period [14].

The results of antibacterial efficacy for high concentrations of *E. coli* by the shaking culture method suggest that GNT-HAp/Col powder has good potential for prevention of infection.

In comparison to the inhibition zone ratio of Ag-containing HAp compact (Ag-HAp) reported by Honda et al. [15], the inhibition zone ratio of the GNT-HAp/Col powder compact at 48 h was larger than that of Ag-HAp. The inhibition zone test also suggested that the antibacterial efficacy of the GNT-HAp/Col would be sufficient up to 48 h. Ideally, an antibiotic-loaded bone cement should release antibiotics over a short period, after which the release should stop, in order to prevent subinhibitory concentrations thereafter, so as not to induce bacterial resistance [16]. For the antibiotic-impregnated bone cement, Palacos^®^ bone cement also released gentamicin over a short period in the in vitro study and showed good antibiotic activities. From this effective theory, an anti-bacterial non-biodegradable implant, the AG-PROTEX^®^, also focused on initial antibacterial properties. However, bacteria from inside of our bodies may also form a biofilm on the surface of implants. Furthermore, HAp/Col was expected to be less effective as a scaffold for forming biofilms because resorption of HAp/Col by osteoclasts starts at 5 days after implantation. Thus, antibiotic efficacy of the GNT-HAp/Col is considered as being sufficient for the prevention of IAI.

GNT was added as an antibiotic to cell culture medium at the recommended concentration of 0.05 mg/mL instead of the generally used penicillin and streptomycin. Concentration of GNT in the present cell culture medium supplemented with the GNTEL was at most 0.038 mg/mL, which is lower than the recommended concentration to avoid obvious harm to cells.

## 4. Materials and Methods

All procedures described in Section 4.1, Section 4.2, and Section 4.3 were carried out in a class 10,000 clean-room at 25 °C and 50% humidity.

### 4.1. Preparation of HAp/Col and HAp/Col Powder

HAp/Col with a HAp/Col mass ratio of 4:1 was synthesized by the simultaneous titration method [7]. Briefly, Ca(OH)_2_, which was prepared by hydration of CaO obtained from heat decomposition of CaCO_3_ (alkaline analysis grade, Wako Pure Chemicals Inc., Osaka, Japan), and orthophosphoric acid (reagent grade, Wako Pure Chemicals Inc., Osaka, Japan) aqueous solution containing porcine dermal type-I atelocollagen (biomaterial grade, Nitta Gelatin Inc., Yao, Japan) were simultaneously titrated into a reaction vessel, in which water was previously added, via tube pumps, maintaining the pH and temperature of the solution in the reaction vessel at 9 and for 37 °C, respectively. The amounts and concentrations of the starting materials were decided by calculation that the resulting HAp/Col became the target ratio under the ideal reaction [7].

The obtained HAp/Col was compacted to a disk at 32 mm in diameter and 2 mm in thickness by a uniaxial pressing at 20 MPa for 18 h to squeeze water out of the obtained HAp/Col [17]. The HAp/Col disk was frozen and freeze-dried at −20 °C, crushed by hand, and sorted into 100–212 μm by sieving. The HAp/Col powder was dehydrothermally cross-linked at 140 °C for 12 h in vacuo. Then, it was stirred in 20 mM CaCl_2_ aqueous solution for 3 days to be saturated for Ca^2+^ adsorption [18], followed by filtering, freeze-drying, and re-sieving to collect the HAp/Col powder 100–212 μm in size. Morphologies of the prepared powder were observed with SEM (JSM-5600, JEOL, Akishima, Japan) at 20 kV acceleration voltage and 20 mA current. The specific surface area of the powder was calculated by the BET method from nitrogen adsorption data obtained with Belsorp II (MicrotracBEL Corp., Osaka, Japan).

### 4.2. Adsorption of GNT on HAp/Col Powder

Gentamicin sulfate (Wako Pure Chemical Co., Osaka, Japan) was chosen as a model antibiotic because it has many advantages such as low cost, effectiveness against most Gram-negative microorganisms, and low risk of generating resistant bacteria, and it is currently used for treatment of IAI by introduction into Palacos. The GNT stock solution was prepared by dissolution of 10 mg of the GNT powder with 10 mL of PBS (Gibco by Life Technologies, Thermo Fisher Scientific KK., Tokyo, Japan). Experimental GNT solutions at a concentration of 50, 100, or 250 μg/mL were prepared by serial dilution of the GNT stock solution with PBS. A total of 10 mg of the HAp/Col powder and 1 mL of the experimental GNT solution were added into a 1.5 mL centrifuge tube and mixed at 4 °C for 24 h with a rotating tube shaker at a rotation rate of 60 rpm. After mixing for 1, 5, 10, 15, 30, 60, 120, 180, 360, 720, or 1440 min, supernatant (500 µL) was collected after centrifugation at 12,000 rpm at 4 °C for 1 min, followed by storage at 4 °C until the quantitative analysis of the GNT (described in Section 4.4). The experiment was performed in triplicate.

### 4.3. Desorption of GNT from GNT-HAp/Col Powder

Gentamicin sulfate-loaded HAp/Col powder was prepared for GNT desorption property measurement. A total of 300 mg of HAp/Col powder and 30 mL of 10 mg/mL GNT solution were mixed at room temperature for 24 h, and the powder phase was collected by centrifugation at 1200 rpm at 25 °C for 5 min, followed by aspiration of supernatant and freeze-drying of remaining powder at −20 °C for 48 h. After GNT-loading, mean powder size of GNT-HAp/Col powder was measured by the line-intercept method.

A total of 100 mg of GNT-HAp/Col powder was added into 10 mL of PBS in a 15 mL centrifuging tube, and was then placed in an incubator at 37 °C without any agitation. Then, 1 mL of supernatant was collected at days 1, 3, 5, 7, and 9. After each collection, 1 mL of fresh PBS was added to compensate for the decrease in liquid amount. Collected samples were stored at 4 °C until quantitative analysis of GNT (described in Section 4.4). The experiment was performed in triplicate.

### 4.4. Quantification of GNT

The amount of GNT in solutions was measured by the Zhang [19] modified Sampath’s [20] method using o-phthaldialdehyde (OPA; Nakarai Tesque, Inc., Kyoto, Japan) solution. Briefly, 0.5 mL of OPA was thoroughly mixed with 0.5 mL of sample and 0.5 mL of 2-propanol (Nakarai Tesque, Inc., Kyoto, Japan), and was incubated at room temperature for 30 min. After incubation, absorbance of the mixture’s wavelength at 332 nm was measured with a UV-VIS spectrometer (V-650, JASCO Co., Tokyo, Japan).

### 4.5. Antibacterial Efficacy Test

A shaking culture method and inhibition zone assay were performed to evaluate the antibacterial efficacy of the extract and the in situ-leached GNT from the GNT-HAp/Col powder, respectively. *E. coli*, a common bacteria that causes infections, was used as the test bacterium. Suspension of *E. coli* was prepared using LB medium (Nihon Purechemical Co., Ltd., Tokyo, Japan) to recommend concentrations by JIS Z2801 (antibacterial products—test for antibacterial activity and efficacy) for each test condition.

#### 4.5.1. Preparation of Samples for Antibacterial Efficacy Test

Extraction liquid of GNT and compacted GNT-HAp/Col powder were prepared for each test method using GNT-HAp/Col powder, which was prepared as described in Section 4.3. Next, 15 mL centrifuge tubes containing 100 mg of GNT-HAp/Col powder and 10 mL of PBS were placed in an incubator at 37 °C without any agitation. After 1 or 5 day incubation, 8 mL of supernatant, GNT extraction liquid (respectively GNTEL 1 and GNTEL 5), was collected and sterilized by filtration. A compact disk of 12 mm in diameter and 1 mm in thickness was prepared by a uniaxial pressing of 100 mg of the GNT-HAp/Col powder at 50 MPa for 5 min, and was sterilized by ethylene oxide gas.

#### 4.5.2. Shaking Culture Method

The lowest, 1 × 10^5^ CFU/mL, and highest, 1 × 10^7^ CFU/mL, *E. coli* concentrations described in the JIS were chosen for the test. A test mixture containing 1 × 10^5^ (L) or 1 × 10^7^ CFU/mL (H) *E. coli* was prepared in a 50 mL centrifuge tube by mixing of 1 mL of GNTEL 1 or 5, and 9 mL of LB medium containing appropriate concentrations of *E. coli*. The mixture was then incubated in a rotary shaker at 180 rpm in the dark at 37 °C for 18 h. After 18 h incubation, the concentration of *E. coli* was quantified with a UV/VIS spectrophotometer (GeneQuant 100, Biochrom. Ltd., Cambridge, UK). Mixed solutions that were prepared with 1 mL of PBS instead of GNTEL were used as controls.

#### 4.5.3. Inhibition Zone Assay

*Escherichia coli* at 1 × 10^7^ CFU were uniformly distributed on the surface of the LB agar plate. The compact was then placed on the center of the LB agar plate, and 500 μL LB medium was added from the top of the compact, followed by incubated at 37 °C in the dark. After 24 or 48 h of incubation, areas of the inhibition zones forming around the compact were measured with ImageJ (Ver. 1.52a for Windows 10, National Institute of Health, Bethesda, MD, USA) from three different JPEG images of 3024 × 3024 pixels taken with an iPhone SE (MLM62J/A, Apple Inc., USA). A compact of HAp/Col powder was used as a control. Inhibition zone ratios as given by the following formula were used:

(2) $$\mathrm{Inhibition}\;\mathrm{zone}\;\mathrm{ratio}=\frac{Z1-Z2}{Z2}$$

where *Z*1 and *Z*2 are the area of the inhibition zone and powder compacts, respectively [15].

### 4.6. Cytotoxicity Test

Cytotoxicity of the GNT-HAp/Col powder was evaluated by addition of GNTEL to culture medium using the MG-63 osteoblastic cell line derived from human osteosarcoma. Culture medium was Dulbecco’s modified Eagle’s medium (DMEM, Sigma-Aldrich, Inc., St. Louis, MO, USA) supplemented with 10% fetal bovine serum (FBS; Sigma-Aldrich, Inc., St. Louis, State of Missouri, USA) and 10% GNTEL collected on days 1, 3, (GNTEL 3) or 5. To clarify the effects of GNT leached from the GNT-HAp/Col, no antibiotics were added into the culture medium. Cell culture medium prepared with 10% PBS instead of GNTEL was used as a control.

One milliliter of MG-63 cell suspension at 1 × 10^4^ cells/mL was prepared with each medium and was seeded into each well of a 12-well tissue culture plate (12-well Clear Flat Bottom TC-treated Multiwell Cell Culture Plate, FALCON, Becton Dickinson Co., Franklin Lakes, NJ, USA). Medium was changed every 2 days, and the number of cells was counted with a hemocytometer on days 1, 3, 5, and 7.

## 5. Conclusions

HAp/Col powder adsorbed at most at 3.4 ± 0.7 mg/g GNT according to Langmuir’s model. The GNT-HAp/Col powder demonstrated burst desorption of all GNT into PBS within 3 days; however, for rapidly biodegradable bone void fillers, burst desorption of antibiotics would be sufficient for prevention of infection, as bacteria cannot increase in population in this environment until complete decomposition of the bone void fillers, and this would avoid generation of resistant bacteria. The leached GNT showed sufficient antibacterial efficacies in both the shaking culture method and the inhibition zone assay using *E. coli*. Furthermore, GNTELs from the GNT-HAp/Col powder showed no cytotoxicity for MG-63. Consequently, GNT-HAp/Col is a good candidate for a bioresorbable bone void filler with prevention of implant-associated infection.

## Figures and Tables

**Table 1:** Final concentration of *E. coli* after the shaking culture test.

Label	L-1	L-5	H-1	H-5	L-C	H-C
Final concentration of *E. coli* (± S.D.)	0	0	9.9 × 10^6^ ±4 × 10^5^	1.0 × 10^7^ ±4 × 10^5^	3.5 × 10^9^ ±3 × 10^7^	3.4 × 10^9^ ±2 × 10^7^
